# The Effect of Hemoglobin Concentration on Hyperbaric Oxygen and Non-hyperbaric Oxygen in the Treatment of Hypertensive Intracerebral Hemorrhage After Operation at the High Altitude

**DOI:** 10.3389/fnhum.2022.834427

**Published:** 2022-06-30

**Authors:** Linjie Wei, Chi Lin, Xingsen Xue, Shiju Jila, Yalan Dai, Li Pan, Wei Wei, Guodong Dun, Yong Shen, Taoxi Zong, Jingjing Wu, Yafang Li, Lixia Wu, Jishu Xian, Anyong Yu

**Affiliations:** ^1^Department of Neurosurgery, PLA 956th Hospital, Linzhi, China; ^2^Department of Neurosurgery, First People’s Hospital of Honghe City, Honghe, China; ^3^Department of Neurosurgery, Southwest Hospital, Third Military Medical University (Army Medical University), Chongqing, China; ^4^Medical Imaging Department, General Hospital of Tibet Military Region, Lhasa, China; ^5^Department of Emergency, Affiliated Hospital of Zunyi Medical University, Zunyi, China

**Keywords:** high altitude, puncture and drainage hematoma, hyperbaric oxygen, computed tomography perfusion imaging, hemoglobin concentration

## Abstract

**Background:**

The prognosis of hypertensive intracerebral hemorrhage (HICH) is poor at high altitudes. The objective of this study was to explore whether hyperbaric oxygen (HBO) can improve the results of computed tomography perfusion (CTP) imaging and the neurological function of patients with HICH, and influence the hemoglobin concentration.

**Method:**

The patients with HICH were treated with puncture and drainage. Twenty-one patients (51.22% of 41 patients in total) were treated with HBO after the operation, and the other patients received conventional treatment. CTP was performed twice, and all indices were measured. Scatter plots were used to determine the effect of hemoglobin concentration on CTP imaging. Receiver operating characteristic (ROC) curves were plotted to analyze the effects of hemoglobin concentration and hematoma volume on recovery results. The patients were followed up for 6 months.

**Results:**

Forty-one patients with HICH were treated with puncture and drainage. In total, 21 were treated with HBO after the operation, and 20 received conventional treatment as the control group. No significant differences in the CBV and CBF values of the two groups were noted before treatment. After 10 days, the values of CBV and CBF in the HBO group were significantly higher than those in the control group. A scatter diagram showed there was no significant in the HBO group, but significant correlation for the CBV and CBF values in the control group’s hematoma center and margin. The ROC curves showed that hematoma volume had an influence on prognosis of the control group. The Glasgow Coma Scale (GOS) scores of the HBO group were significantly higher than those of the control group (*p* < 0.05).

**Conclusions:**

HBO therapy can improve the postoperative CBV and CBF values of patients with HICH and ameliorate their prognoses. There was no significant correlation between HBO group and hemoglobin concentration on admission.

## Introduction

At high altitude regions the hemoglobin concentrations will increase because of hypoxia. However, if the hemoglobin levels increase too much, the viscosity of the blood will increase, and blood flow will be slowed. Thus the hypoxia of brain tissue will further accelerate. Hyperbaric oxygen (HBO) therapy can effectively improve the partial pressure and dispersion rate of blood oxygen, thus leading to nerve function recovery (Li et al., 2017). The literature reports that HBO can significantly improve the recovery of neurological function after cerebral hemorrhage (Peng et al., 2014; Wang et al., 2020). High-altitude areas are more prone to hypertensive cerebral hemorrhage (HICH) than low-altitude areas. Due to the lack of oxygen in high-altitude areas, functional recovery after cerebral hemorrhage is poor (Zhu et al., 2015; Wei et al., 2019). Studies have in particular shown, that there is decreased cerebral blood flow (CBF) after craniocerebral injury at high altitudes (Yu et al., 2014). At present, an effective method to improve self-reliance after cerebral hemorrhage is lacking. It is not clear whether HBO can improve the CBV and thus ameliorate certain neurological functions. In addition, it remains elusive, whether there would be an effect of the hyperbaric or non-hyperbaric oxgen on the hemoglobin concentration in the treatment of hypertensive intracerebral hemorrhage. Therefore, our team conducted a retrospective analysis on the use of HBO in high-altitude areas.

## Materials and Methods

### Patient Selection

**FIGURE 1:**
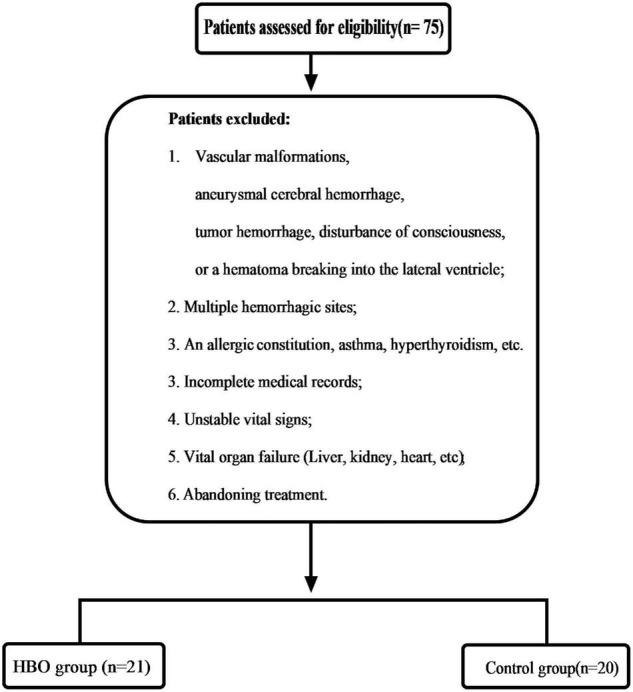
A flow chart for the identification process of eligible patients.

This study was approved by our local ethics committee. These case data were obtained retrospectively. From February 2018 to December 2020, the patients who had HICH as determined by computed tomography (CT) (GE Lightspeed 64, United States) scans and related examinations were enrolled. The selection criteria were as follows: (1) History of hypertension; (2) Altitude (1,500–4,000 m) (2,873.45 ± 745.99 m); (3) Time spent at these altitudes ≥ 5 years; (4) Hematoma volume 30–50 ml; (5) Good liver and kidney function; (6) Stable vital signs; (7) Hypertensive basal ganglia hemorrhage; and (8) Complete case data. Patients were excluded according to Figure 1. Twenty-one patients were treated with HBO after the operation (HBO group), and the other patients received conventional treatment (Control group) (Figure 1). The relevant data of the patients, including sex, age, medication history, hematoma size, hemoglobin concentration, and Glasgow Coma Scale (GCS), were collected and recorded in detail on admission. When the patient were admitted to the hospital, we asked the medical history. If the patient had used anticoagulant and antiplatelet drugs, we tested the patient’s coagulation function. If the patient were seriously affected by coagulation, we treated it conservatively and evaluated that it would not have a great impact on coagulation. We also conducted surgical treatment. All data were independently and blindly reviewed by two senior neurosurgeons.

### Therapeutic Method

**FIGURE 2:**
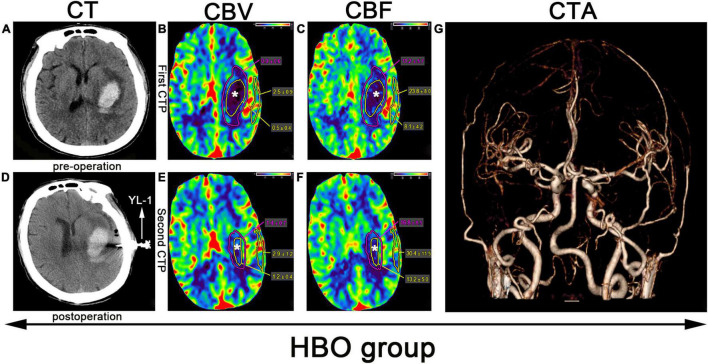
CTP scan from a patient in the HBO group: **(A)** Preoperative left basal ganglia hemorrhage. **(B,C,E,F)** The yellow line denotes the hematoma area, the white star (*) denotes the hematoma center, the purple line denotes the hematoma margin, and the outer layer yellow line denotes the cortical area of the hematoma. **(D)** Application of a YL-1 puncture needle after puncture and drainage. **(G)** Normal CTA.

**FIGURE 3:**
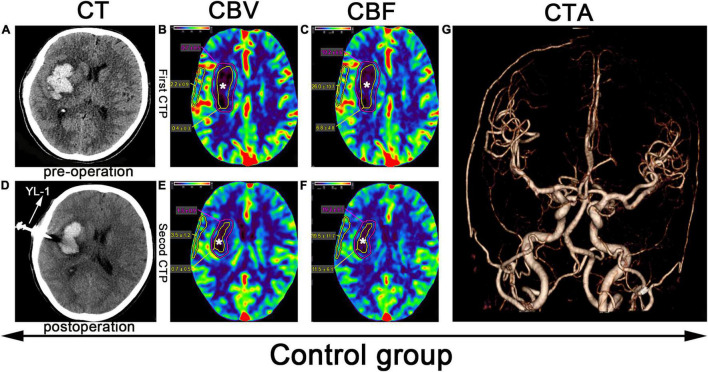
CTP scan from a patient in the control group: **(A)** Preoperative right basal ganglia hemorrhage. **(B,C,E,F)** The yellow line denotes the hematoma center area, the white star (*) denotes the hematoma center, and the outer layer yellow line denotes the cortical area of the hematoma. **(D)** Application of a YL-1 puncture needle after puncture and drainage. **(G)** Normal CTA.

Puncture and drainage hematoma: The largest hematoma plane was selected as the positioning point for drilling (Figures 2A, 3A). All patients were placed under local anesthesia, and a YL-1 puncture needle (Beijing Wan Fu Tie Medical Apparatus Co., Ltd., China) was used for drilling after selecting the positioning point (Xia et al., 2019; Figures 2D, 3D). After puncture and drainage, CT examination revealed that the hematoma was enlarged, and a craniotomy was performed to remove the hematoma. After 3–5 days of drainage, head CT was repeated and indicated that the hematoma had been reduced by 50–100%. The drainage tube was removed. Then, the patients in the HBO group were treated with HBO (100% oxygen at 2.2 ATA for 60 min for 10 days). In the control group, all treatments were the same, except HBO treatment was lacking. If the hematoma expanded more than 50 ml, we performed craniotomy to remove the hematoma. If the enlarged hematoma was less than 50 ml, but the GCS score decreased by 2 points, craniotomy was also considered.

### Imaging Data Measurement

Before puncture and drainage, CT angiography (CTA) was completed, and the result was normal (Figures 2G, 3G). CT perfusion (CTP) imaging was performed after written informed consent was obtained from the patients or their relatives. CTP imaging was performed twice. The first imaging scan was performed before HBO treatment for the HBO group and before standard treatment for the control group (Figures 2B,C, 3B,C); both groups underwent scans on the same number of days prior to the start of their respective treatments. The second CTP imaging scan was performed 10 days after HBO treatment for the HBO group and 10 days after the corresponding treatment for the control group (Figures 2E,F, 3E,F). A high-pressure syringe was used to inject iohexol (Yangtze River Pharmaceutical Group Co., Ltd., Production) through the anterior elbow vein (17.5 g in 50 ml, flow rate: 5 ml/s) (Wang et al., 2017). In the hematoma center, different regions of interest (ROIs) for the hematoma margin (defined as the area around the hematoma with a CT value at least 5 U less than of normal brain tissue or the area 1 cm away from the hematoma if the CT value was less than 5 U that of the brain tissue) and cortical area on the hematoma side (measuring the same length as the hematoma and edema) were selected for qualitative analysis. The mean perfusion parameters (cerebral blood volume (CBV) and (CBF) were calculated. The above parameters were also calculated for the control group. After 10 days, CTP imaging was performed again, and the parameters were recalculated. The above parameters were transferred to 4D Brain Perfusion (Vitrea, Japan) for ROI analysis. The formula ABC/2 was used to calculate the volume of the hematoma (Kothari et al., 1996). All data were independently and blindly reviewed by two senior neurosurgeons.

### Evaluation and Follow-Up

The CBV and CBF values of the two groups were measured and compared. A scatter diagram was plotted to demonstrate the relationship between the hemoglobin concentration on admission and the second set of CBV and CBF values. Receiver operating characteristic (ROC) curves were used to measure the cutoff values at which hematoma volume and hemoglobin concentration predicted a good outcome and the corresponding specificities and sensitivities. After a 6-month follow-up, the patients were scored using the Glasgow Outcome Scale (GOS). The scoring criteria were as follows: Good recovery (5), Moderate disability (4), Severe disability (3), Vegetative state (2), and Death (1). The patients were divided into 2 outcome groups: a good outcome group, which was defined as independent (GOS scores, 4-5), and a poor outcome group, which was defined as dependent (GOS scores, 3-1) (Dunatov et al., 2011).

### Statistical Analysis

All analyses were performed using SPSS 19 (IBM Corp., Armonk, NY, United States). Normally distributed data are expressed as the mean ± standard deviation. Quantitative data were analyzed between the two groups using separate *T*-tests and analysis of variance. Differences were considered significant at *p* < 0.05. A scattergram with a regression line was generated to determine the association between the hemoglobin concentration and CBV and CBF. ROC curve analysis was used to investigate the diagnostic relationship between risk factors and a good outcome. The Youden index was used to determine the optimal cutoff value as previously described (Wei et al., 2020).

## Results

### Participant Characteristics

A total of 41 patients were included in the study, of whom 21 received HBO treatment and 20 received conventional therapy. No significant differences in the basic characteristics of the two groups were noted (Table 1). Partial hematomas could not be extracted from two patients in the HBO group and three patients in the control group, resulting in success rates of 90.48 and 85%, respectively. The hematoma could not be extracted for several patients in the two groups, but their conditions were stable. Later, treatment was performed, and these data on the two groups were included. There were no significant differences in the success rate of the two groups (*p* > 0.05). No intracranial infection was found among the 41 patients.

**TABLE 1:** Patient characteristics of the HBO and control groups.

Characteristics	HBO group (n) 21	Control group(n) 20	t/χ^2^	*P*-value
Age (y) (Mean ± SD)	53.35±10.85	50.75±7.31	0.721	0.477
Men (n)	15	13	0.196	0.744
**Ethnicity**				
Ethnic minorities (n)	12	9	0.605	0.538
Han Chinese (n)	9	11		
Hematoma volume (ml) (Mean ± SD)	39.43±5.95	39.55±6.48	0.063	0.950
**Comorbidities**				
Diabetes (n)*	3	4		0.697
Heart disease (n)	6	5	0.067	0.796
Pulmonary disease (n)*	4	5		0.719
Other diseases (n)	5	5	0.008	0.929
GCS, median (IQR)^Δ^	12 (7,15)	11(6,14)		
**Time from onset to admission**				
(h) (Mean ± SD)	4.47±1.67	3.95±1.93	0.936	0.355
**Hemoglobin concentration**				
(g/L) (Mean ± SD)	179.67±18.25	178.10±23.28	0.24	0.811

*Fisher’s exact test; y, year; GCS, Glasgow coma scale; h, hour; ^Δ^Mann–Whitney U test.

### CTP Imaging and Follow-Up Results

**FIGURE 4:**
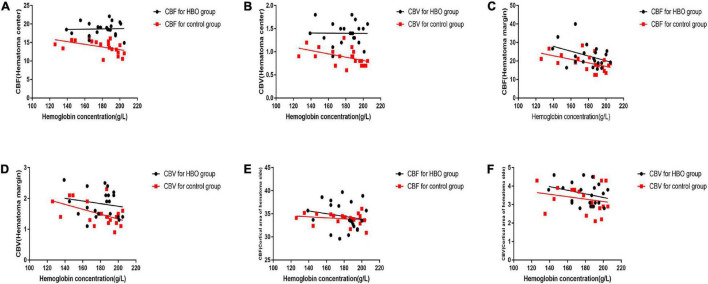
A scatter diagram of black dots are depicted for the association between hematoma volume and the second set of CTP parameters in the HBO group. The relationship was not linear for the CBV and CBF values of the hematoma center, hematoma margin and cortical area of the hematoma **(A–F)**. A scatter diagram of red box are depicted for the correlation between hematoma volume and the second set of CTP parameters in the control group. The relationship was linear for the CBV and CBF values of the hematoma center **(E,F)** nor for the CBV and CBF values of hematoma margin **(A–D)**. However, no significant linear correlation was observed for the CBV and CBF values of the cortical area of the hematoma **(E,F)**.

**FIGURE 5:**
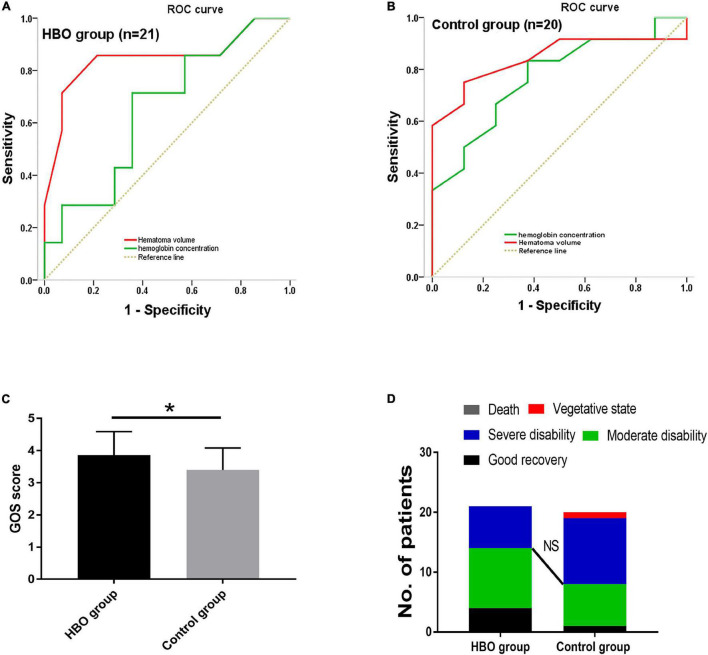
ROC curve analysis was used to determine the cutoff values for hematoma volume and hemoglobin concentration in predicting the outcome after surgery. Specificity and sensitivity were used as measures of outcome accuracy. Analyses were performed in the HBO group **(A)** and the control group **(B)**. The outcome was estimated using the area under the ROC curve (AUC). The GOS scores of the HBO and control groups were obtained 6 months after cerebral hemorrhage and were significantly higher in the HBO group than in the control group **(C)**. However, NS indicates that there was no significant difference in the percentage of good outcomes between the groups **(D)**. Statistically significant differences, indicated by *P < 0.05.

No significant differences in the CBV and CBF values obtained from the first CTP scans were noted between the two groups (Table 2). CBV and CBF values of the hematoma centers and margins from the second scan in the HBO group were significantly higher than those in the control group (Table 2). In both groups, we analyzed the correlation between the hemoglobin concentration on admission and the CBV and CBF values extracted from the second CTP scan by generating a scatter diagram. The correlations between the hemoglobin concentration on admission and the CBV and CBF values from the hematoma center, margin and cortical area on the hematoma side were not significant in the HBO group (hematoma center: CBV: *R*^2^ = 0.00, *p* = 0.96; CBF: *R*^2^ = 0.00, *P* = 0.86; hematoma margin: CBV: *R*^2^ = 0.03, *p* = 0.44; CBF: *R*^2^ = 0.15, *p* = 0.07; cortical area on the hematoma side: CBV: *R*^2^ = 0.09, *p* = 0.19 and CBF: *R*^2^ = 0.03, *p* = 0.45) (Figures 4A–F). The correlations between the hemoglobin concentration on admission and the CBV and CBF values from the hematoma center, margin were significant in the control group (hematoma center: CBV: *R*^2^ = 0.21, *p* = 0.04 and CBF: *R*^2^ = 0.22, *p* = 0.04; hematoma margin: CBV: *R*^2^ = 0.24, *p* = 0.03 and CBF: *R*^2^ = 0.25, *p* = 0.02) (Figures 4A–D) group, but the correlations with the CBV and CBF values from the cortical area on the hematoma side were not significant in the control group (CBV: *R*^2^ = 0.04, *p* = 0.38 and CBF: *R*^2^ = 0.04, *p* = 0.39) (Figures 4E,F). ROC curve analysis showed that the AUC was 0.847 for hematoma volume and 0.653 for hemoglobin concentration (Table 3 and Figure 5A) in the HBO group. Similarly, the AUC was 0.844 for hematoma volume and 0.771 for hemoglobin concentration (Table 3 and Figure 5B) in the control group. ROC curve analysis showed that the specificity and sensitivity of hematoma volume were higher than those of hemoglobin concentration in the two groups (Figures 5A,B). The follow-up duration was 6 months. The GOS score of the HBO group was significantly higher than that of the control group (Figure 5C) (*p* < 0.05). However, there was no significant difference in the percentages of good outcomes between the two groups (Figure 5D).

**TABLE 2:** Comparison of perfusion parameters in the central, marginal and cortical areas of the hematoma.

Observation area	FCBV (mL⋅100 g^–1^)		FCBF (mL⋅100 g^–1^⋅min^–1^)		SCBV (mL⋅100 g^–1^)		SCBF (mL⋅100 g^–1^⋅min^–1^)	
	HBO group	Control group	HBO group	Control group	HBO group	Control group	HBO group	Control group
Hematoma center	0.66 ± 0.21	0.58 ± 0.19^Δ^	9.25 ± 2.05	8.83 ± 1.92^Δ^	1.40 ± 0.24	0.89 ± 0.18***	18.68 ± 1.81	13.86 ± 1.82***
Hematoma margin	1.01 ± 0.38	0.96 ± 0.27^Δ^	16.70 ± 0.52	17.28 ± 1.83^Δ^	1.88 ± 0.44	1.50 ± 0.37**	22.76 ± 6.02	19.18 ± 4.48*
Cortical area of the hematoma	2.74 ± 0.08	2.52 ± 0.49^Δ^	27.86 ± 2.88	27.17 ± 4.58^Δ^	3.59 ± 0.60	3.21 ± 0.74^Δ^	34.47 ± 3.05	33.94 ± 1.24^Δ^

FCBV, FCBF obtained from the first CTP scan; SCBV, SCBF obtained from the second CTP scan. (HBO group vs Control group:Δ: p > 0.05; *p < 0.05, **p < 0.01, ***p < 0.001).

**TABLE 3:** Indices of lowest relative factors for predicting good outcome.

Indices	HBO group (*n* = 21)		Control group (*n* = 20)	
	Hematoma volume	Hg	Hematoma volume	Hg
Cutoff value (%)	64.28%	35.71%	58.33%	45.83%
AUC	0.847	0.653	0.844	0.771
95% CI	(0.635–1.000)	(0.405–0.901)	(0.663–1.000)	(0.561–0.980)
Sensitivity (%)	85.71%	71.42%	58.33%	83.33%
Specificity (%)	78.58%	64.29%	100%	62.5%
*p*-Value	<0.05	>0.05	<0.05	<0.05

*Hg: hemoglobin concentration.*

## Discussion

HBO treatment for HICH was conducive to the improvement of neurological function. The results showed that the CBV and CBF values and the GOS scores of the HBO group were significantly higher than those of the control group. In addition, the volume of the hematoma on admission was negatively correlated with the CBV and CBF values extracted 10 days later. And intriguingly, hyperbaric oxygen therapy can reduce the effect of hemoglobin concentration on CBV and CBF. The larger the hematoma volume was, the longer it took for CBV and CBF to return to normal. HBO therapy has demonstrated a good effect on postoperative HICH at low altitudes. However, the hemoglobin concentration increases at high altitudes, and the pathophysiology is different from that at low altitudes (Yu et al., 2014; Cavalcante and Ormond, 2018). HBO treatment can transport oxygen to hypoxic tissue through higher-than-normal atmospheric pressure, reduce brain edema, improve oxygen diffusion ability and ameliorate nerve cell function (Wu et al., 2018). In addition, studies have shown that intracerebral hemorrhage is essentially an inflammatory reaction (Weng et al., 2015). HBO reduces inflammatory reactions and improves neurological function (Qian et al., 2017). Moreover, elevated hemoglobin levels increase blood viscosity and reduce oxygen delivery, thus leading to insufficient blood supply and even cerebral infarction (Chen et al., 2017). Our team reviewed the case results and found that HBO treatment could significantly improve the CBV and CBF values extracted from the hematoma center and margin at high altitudes. The greater the CBV and CBF values were, the better the circulation of the brain tissue. The main reason is that HBO alleviates the hypoxic condition of compressed brain tissue, ameliorates the function of nerve cells, and reduces tissue ischemia and hypoxia (Matchett et al., 2009), thus enhancing CBV and CBF. The preoperative size of the hematoma had an impact on the postoperative CBV and CBF values. Our results showed that larger hematomas were associated with worse CBV and CBF recovery, greater intracranial pressure, heavier edemas, and worse neurological function recovery. Therefore, through CTP examination, it was clear that HBO improved the postoperative CBV and CBF values and thus neurological function. Through HBO treatment and improved brain tissue perfusion and neurological function, the GOS scores of the HBO group increased. However, there was no difference in the percentage of patients with a good outcome, which might be due to the small number of patients, making the results unclear.

## Conclusion

HBO could significantly ameliorate CBV and CBF and improve the recovery of neurological function. A large preoperative hematoma might affect CBV and CBF recovery and HBO can reduce the effect of hemoglobin concentration on CBV and CBF.

### Limitations

This study was limited by the case load, its retrospective design, and the fact that the analysis was restricted to basal ganglia hemorrhages. These limitations might restrict the generalizability of our findings.

## Data Availability Statement

The raw data supporting the conclusions of this article will be made available by the authors, without undue reservation.

## Ethics Statement

This study was approved by PLA 956th Hospital and First People’s Hospital of Honghe City Ethics Committee. The patients/participants provided their written informed consent to participate in this study. Written informed consent was obtained from the individual(s) for the publication of any potentially identifiable images or data included in this article.

## Author Contributions

AY designed the study. LW, CL, XX, SJ, YD, LP, WW, GD, YS, TZ, JW, YL, and LW collected the case data. LW and JX revised the manuscript to the final draft. LW contributed to the important discussion and interpretation of the results. All authors read and approved the final version of this manuscript.

## Conflict of Interest

The authors declare that the research was conducted in the absence of any commercial or financial relationships that could be construed as a potential conflict of interest.

## Publisher’s Note

All claims expressed in this article are solely those of the authors and do not necessarily represent those of their affiliated organizations, or those of the publisher, the editors and the reviewers. Any product that may be evaluated in this article, or claim that may be made by its manufacturer, is not guaranteed or endorsed by the publisher.

## Abbreviations

CT: Computed tomography
GCS: Glasgow Coma Scale
CTP: Computed tomography perfusion
GOS: Glasgow Outcome Scale
ROI: Region of interest
CBV: Cerebral blood volume
CBF: Cerebral blood flow
CTA: Computed tomography angiography
HICH: Hypertensive cerebral hemorrhage
HBO: Hyperbaric oxygen.
